# Lidocaine constant rate infusion in isoflurane anesthetized neonatal foals

**DOI:** 10.3389/fvets.2023.1304868

**Published:** 2024-01-17

**Authors:** Carlotta Lambertini, Francesca Spaccini, Alessia Mazzanti, Alessandro Spadari, Aliai Lanci, Noemi Romagnoli

**Affiliations:** ^1^Department of Veterinary Medical Sciences, Alma Mater Studiorum, University of Bologna, Bologna, Italy; ^2^Private Practitioner, Bologna, Italy

**Keywords:** lidocaine, anesthesia, analgesia, constant rate infusion, horses, partial intravenous anesthesia, sparing effect

## Abstract

**Introduction:**

In horses, lidocaine infusion is administered intraoperatively for analgesia and for a reduction of inhalant anaesthetic requirement. The objective of the study was to describe the anaesthetic effects of lidocaine infusion in isoflurane anaesthetised foals.

**Methods:**

Twelve foals (<3 weeks old) undergoing surgery were included in the study (LIDO group). Foals were premedicated with midazolam and butorphanol IV, anaesthesia was induced with ketamine and propofol IV and maintained with isoflurane. Lidocaine was administered intraoperatively at 0.05 mg/kg/min. Also, the anaesthetic records of 11 foals in which lidocaine was not administered intraoperatively were retrospectively evaluated and they were considered as a historical control group (HC). Heart rate (HR), mean arterial pressure (MAP) and fraction of expired isoflurane were monitored continuously. Time of extubation, time to reach sternal recumbency and standing were recorded. The quality of recovery was assessed.

**Results:**

HR decreased in both groups compared with baseline values and intraoperatively the differences were statistically significant (*p* = 0.01 and *p* = 0.03 respectively in the LIDO and HC groups). Intraoperatively the HR was significantly lower in the LIDO group (71.2 ± 13.4 bpm) compared with the HC group (87.1 ± 17.7 bpm) (*p* = 0.0236). The number of foals requiring inotropic support (LIDO *n* = 7 and HC *n* = 9) was not statistically associated with the treatment group (*p* = 0.371). The extubation time, the time to reach the sternal recumbency and the quality of recovery did not differ significantly between the two groups (*p* = 0.7 and *p* = 0.6 respectively).

**Discussion:**

In conclusion, in anaesthetised foals the addition of lidocaine does not provide a sparing effect on isoflurane requirement, and it does not interfere with the quality of recovery, however it decreases significantly the HR, which is pivotal in foals for the maintenance of cardiac output and peripheral perfusion. Therefore, a continuous patient monitoring is essential.

## 1 Introduction

Surgical procedures under general anesthesia might be required in neonatal foals in the first week after birth for a variety of reasons, ranging from minor elective procedures to emergency situations, such as septic arthritis, uroperitoneum, trauma, or gastrointestinal surgery (1). Compared with adult horses, foals are at higher risk of perianesthetic death; therefore, the anesthetic management of these animals requires special consideration (2, 3). In fact, compared to adults, foals from birth to 1 month of age are characterized by an immature sympathetic nervous system, low myocardial compliance, and different pharmacokinetics and pharmacodynamics of anesthetics (2). As a result, anesthetic agents that are administered when there are pathological conditions, such as dehydration, sepsis, and electrolyte abnormalities, may exert a greater impact on cardiovascular function (4). However, it is also well recognized that, both in humans and animal species, infants possess mature pain pathways, and in adults, appropriate pain control guarantees a better outcome after surgical procedures (2, 5).

General anesthesia in horses is usually accomplished with inhalant anesthetic agents that lead to a dose-related cardiovascular and respiratory depression (6). Therefore, balanced anesthesia with additional analgesia can minimize the requirement of volatile anesthetics. Previous studies have demonstrated that the intraoperative infusion of lidocaine reduces the minimum alveolar concentration (MAC) of inhalant anesthetics in a dose-dependent manner in humans (7), dogs (8), and horses (9). Dzikiti et al. (9) showed that, in horses, systemic lidocaine allowed a reduction of 25% in the isoflurane required, without a negative effect on the cardiovascular system. Similar results were reported in the study by Rezende et al. (10), in which lidocaine administrated as a bolus of 1.3 mg/kg during a 15-min period, followed by constant rate infusion (CRI) at 0.05 mg/kg/min, provided a mean MAC reduction of 26.7 ± 12%.

To the best of the authors’ knowledge, no reports have been published describing the use of lidocaine infusion in anesthetised neonatal foals. The authors hypothesized that lidocaine would reduce the MAC of isoflurane in anesthetized neonatal foals in order to optimize anesthesia and reduce the cardiopulmonary effects related to volatile anesthetics.

Therefore, this study aimed to describe the anesthetic effects of lidocaine CRI in neonatal foals during general anesthesia.

## 2 Materials and methods

The study was conceived as a clinical trial in which privately owned foals requiring anesthesia were included. The study has been assessed by the Animal Welfare Committee of the University of Bologna (Italy), which has provided a positive ethical-scientific evaluation and confirmed its compliance with national laws on the matter. All the procedures were performed with the owners’ consent.

### 2.1 Experimental group (LIDO)

A group of 12 privately owned foals less than 3 weeks of age admitted to the Veterinary Teaching Hospital of the University of Bologna from March 2020 to April 2021 and underwent surgery were included in the study. Complete blood count and serum biochemistry analyses were obtained upon arrival, and physical examination was carried out before providing anesthesia. The foals were then assigned to the physical status classification of the American Society of Anesthesiologists (ASA).

Foals greater than 3 weeks of age, foals in which the scheduled anesthetic protocol was not feasible, and those in which intraoperative euthanasia was performed were excluded from the study.

Before anesthesia, a 16-gauge central venous catheter (LOGICATH™, Smiths Medical Inc.) was placed in the jugular vein by the Seldinger technique using a standard sterile technique after aseptic preparation of the area.

All the foals were premedicated with 0.05 mg/kg of butorphanol (Nargesic, ACME S.r.l., Italy) and 0.06 mg/kg of midazolam (Midazolam B. Braun, Germany) administered intravenously (IV). After 5 min, foals were placed on the preparation table in lateral recumbency, and anesthesia was induced with 1.5 mg/kg of ketamine (Nimatek, Dechra veterinary products s.r.l., Italy) and 0.8 mg/kg IV of propofol (Proposure, Boehringer Ingelheim Animal Health Italia S.p.A., Italy) in order to achieve orotracheal intubation, maintaining the foals in lateral recumbency. After anesthesia induction, the foals were moved to the surgical table and attached to an anesthetic machine with isoflurane in oxygen, which was initially set in order to achieve a fraction of expired isoflurane (F_E_’Iso) of 1.5% (Isoflo, Zoetis Italia S.r.l., Italy), and lidocaine CRI was started at 0.05 mg/kg/min (Lidocaina 2%, Ecuphar Italia S.r.l., Italy). After 15 min, the foals were placed in dorsal recumbency and prepared for surgery. Mechanical ventilation was provided if needed in order to maintain the end-tidal carbon dioxide tension (F_E_’CO_2_) between 35 and 45 mmHg by setting a tidal volume of 10–12 mL/kg and an inspiration to expiration ratio of 1:2.

The cardiovascular variables were evaluated and recorded at 5-min interval throughout the procedure using a calibrated multiparametric monitor (Datex-Ohmeda S/5): heart rate (HR) by means of an electrocardiogram, respiratory rate (*f*R), F_E_’CO2, and F_E_’Iso% using a sidestream calibrated gas analyser, haemoglobin oxygen saturation using a pulse oximeter (SpO2), and body temperature, and systolic pressure, diastolic pressure, and mean arterial pressure (MAP) using a non-invasive method by applying the cuff on the forelimb.

In addition, at 5-min interval, an anaesthetist with more than 2 years of clinical experience assessed the anesthetic depth based on vital signs upon application of the surgical stimulation in order to maintain a surgical anesthetic plane. In the case of nystagmus or a central eye globe position with a concomitant brisk palpebral reflex or spontaneous blinking, the F_E_’Iso was increased by 0.1%. The anesthesia given was judged to be too profound when the eye globe was in a central position, and in the absence of the palpebral reflex, the isoflurane was therefore decreased by 0.1%. An additional bolus of propofol (0.5 mg/kg) was administered IV in the case of the movement of the limbs or the head.

Hypotension was defined as an MAP of <60 mmHg (10). Potential hypotension was treated with dobutamine IV (Dobutamina, Bioindustria L.I.M., Italy) at 0.001–0.01 mg/kg/min or with norepinephrine at 0.0005–0.0015 mg/kg/min (2, 11). Bradycardia was defined as an HR of <50 bpm and was treated with atropine (0.02 mg/kg) IV (Atropina, ATI S.r.l., Italy) (1, 12). A 20% increase in the HR or MAP upon the application of the surgical stimulus was considered indicative of inadequate analgesia. In that case, rescue analgesia was provided with ketamine (0.5 mg/kg) administered IV. Those foals receiving ketamine were excluded from further evaluations. In total, 10 mL/kg/h of Ringer lactate solution (Ringer lattato, B. Braun, Germany) infusion was administered IV throughout the procedure. Glucose supplementation was provided, if necessary, in order to maintain a glucose concentration within the reference range (76–131 mg/dL).

Isoflurane administration and infusions were stopped at the end of the surgical procedure. In those foals in which mechanical ventilation was provided, it was discontinued at the end of the surgical procedure, and the foals were allowed to breathe spontaneously. The foals were then placed in lateral recumbency and moved to the intensive care unit. Here, extubation was performed when the first swallowing reflex was observed. The extubation time and time to reach sternal recumbency were recorded as the time interval from the interruption of inhalant anesthesia. In addition, in those foals not requiring assistance for regaining the standing position, the quality of recovery was assessed using a recovery score previously described for foals (13). Moreover, at extubation, the HR and the *f*R were evaluated and recorded.

### 2.2 Historical control group (HC)

An electronic patient database (software Fenice) was used to identify the foals admitted to the Veterinary Teaching Hospital of the University of Bologna, Bologna, Italy, which underwent surgery between March 2015 and June 2019. The anesthetic records of these 11 foals were collected and analysed. Only those foals in which lidocaine was not administered intraoperatively and received the same anesthetic protocol described for the LIDO group for premedication, induction, and maintenance of general anesthesia were included, and they were considered to be a historical control group (HC). For the HC group, the procedures were performed in the same clinic of the foals of the LIDO group, and the same multiparametric monitor was used for anesthetic monitoring. In detail, all the foals were premedicated with butorphanol (0.05 mg/kg) and midazolam (0.06 mg/kg) IV. General anesthesia was induced with ketamine (1.5 mg/kg) and propofol (0.8 mg/kg) IV in order to achieve endotracheal intubation and was maintained with isoflurane in oxygen, which was initially set to reach F_E_’Iso of 1.5%. The F_E_’Iso during anesthesia was adjusted accordingly with the criteria previously described for the experimental group. Ketamine (0.5 mg/kg) was used as a rescue analgesia and was administered IV when the intraoperative analgesia was considered inadequate, evaluated on the basis of a 20% increase in HR and MAP upon the application of the surgical stimulus, as a common practice in hour clinic.

### 2.3 Statistical analysis

Sample size calculation was performed while hypothesizing that a 20% reduction in the fraction of expired isoflurane would be clinically and statistically significant. The analysis indicated that 11 foals would be necessary in each group with an alpha error of 5% and a study power of 0.8.

Statistical analysis was carried out using computer software (Stata/SE 17.0 for MAC; StataCorp, TX, United States). For statistical purposes, the data collected at baseline, the mean of the intraoperative values, and single evaluation at the extubation time were used. The data were tested for normality using a Shapiro–Wilk test. Normally distributed data were reported as mean and standard deviation and were compared by using Student’s t-test or an ANOVA test followed by a Bonferroni correction which was used for comparison within each group. Non-normal distributions of data were compared using a Mann–Whitney test and were reported as median and range. The nominal data (sex, ASA category, and dobutamine administration) were analysed using the Fisher exact test. A *p*-value < 0.05 was considered statistically significant.

## 3 Results

A total of 23 foals were included in this study. Twelve foals (3 fillies and 9 colts) aged 5.3 ± 4.3 days and weighing 55.1 ± 9.6 kg were included in the LIDO group. Eleven foals (5 fillies and 6 colts) aged 5.6 ± 4.3 days old and weighing 49 ± 8.1 kg were included in the HC group. The age, the weight of the foals, and sex distribution between the two groups did not differ significantly.

The distribution of foals into ASA categories did not differ significantly between the two groups (*p* = 0.5) and is shown in Table 1.

**Table 1:** American Society of Anesthesiologists (ASA) categories (I–V) of the 23 foals included into the study.

Group	ASA category				
	I	II	III	IV	V
LIDO (*n*=)	0	6	4	1	1
HC (*n*=)	0	3	5	3	0
Total (*n*=)	0	11	7	4	1

The foals underwent general anesthesia, and lidocaine was administered intraoperatively in the LIDO group but not in the HC group.

The demographic data of the foals included in the two groups and the diagnoses are shown in Table 2. No differences were found between the groups for age, body weight, sex, breed, and diagnosis. In the LIDO group, anesthesia and surgery lasted for 112.8 ± 43.9 and 89.6 ± 36 min, respectively. In the HC group, the anesthesia and surgery lasted for 121.4 ± 47.5 and 92.3 ± 37.2 min, respectively; the length of anesthesia and surgery did not differ between the two groups (*p* = 0.7 and *p* = 0.9 for anesthesia and surgery, respectively).

**Table 2:** Breed, sex, age, weight, and diagnosis of foals undergoing general anesthesia.

Group	Breed	Sex	Age (days)	Weight (kg)	Diagnosis
LIDO	Italian Saddlebred	M	7	45	Urachal diverticulum
	Standardbred	M	10	80	Omphalophlebitis
	Standardbred	M	4	50	Septic arthritis
	Quarter horse	F	5	43	Uroperitoneum
	Italian Saddlebred	M	7	51	Intestinal intussusception
	Standardbred	F	2	63	Uroperitoneum
	Standardbred	M	16	60	Septic arthritis
	Arabian horse	F	2	47	Intestinal intussusception
	Meticcio	M	2	56	Uroperitoneum
	Arabian Horse	M	1	46	Inguinal hernia
	Italian Saddlebred	M	2	58	Septic arthritis
	Italian Saddlebred	M	2	60	Septic arthritis
HC	Standarbred	M	3	45	Uroperitoneum
	Holland horse	F	3	45	Uroperitoneum
	Standardbred	M	4	50	Uroperitoneum
	Standardbred	M	6	48	Omphalitis
	Arabian horse	M	10	50	Omphalitis
	Standardbred	M	2	62	Meconium impaction
	Haflinger	M	4	40	Uroperitoneum
	Italian Saddlebred	F	4	40	Septic arthritis
	Paint	F	1	43	Meconium impaction
	Standardbred	F	10	65	Septic arthritis
	Quarter horse	F	15	51	Septic arthritis

Lidocaine was administered intraoperatively in the LIDO group but not in the HC group. FL = foal; M = male; F = female.

**Figure 1:**
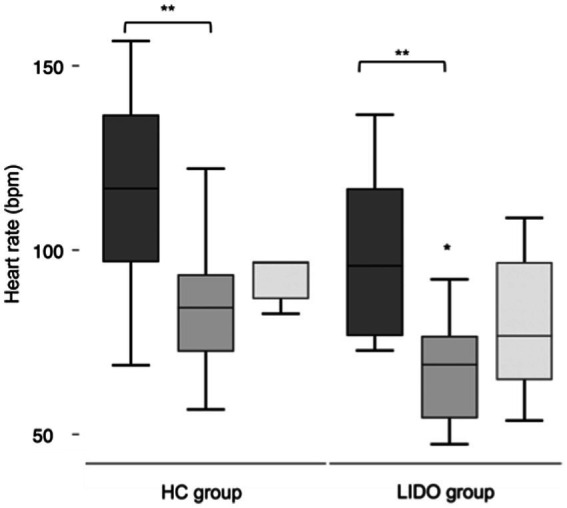
Box and whisker plots for heart rate in isoflurane anesthetized foals. Lidocaine was administered intraoperatively in the LIDO group but not in the HC group. Data were collected at baseline (dark grey), intraoperatively (middle grey), and at the recovery (light grey). The whiskers represent the range of values, the boxes represent the interquartile range, and the lines within each box represent the median value for the heart rate. *Statistically significant between groups. **Statistically significant different compared with baseline.

At the baseline, the mean HRs did not differ significantly between the two groups (*p* = 0.1) and were 102.8 ± 22.8 bpm in the LIDO group and 118.5 ± 24.6 bpm in the HC group. Intraoperatively and at the recovery, the HR decreased in both groups if compared with the baseline values; however, only intraoperatively, the differences were statistically significant (*p* = 0.01 and *p* = 0.03, respectively in the LIDO and HC). Intraoperatively, the HR was significantly lower in the LIDO group (71.2 ± 13.4 bpm) if compared with the HC group (87.1 ± 17.7 bpm) (*p* = 0.02), while the mean MAP was 69.8 ± 9.2 and 62.4 ± 9.7 in the LIDO and HC groups, respectively, without statistically significant differences between the two groups (*p* = 0.08). At the recovery, the mean HRs did not differ significantly between the two groups (*p* = 0.09) and were 84.6 ± 6.8 bpm in the LIDO group and 104.7 ± 27.9 bpm in the HC group. The results of the HR in the two groups are represented in Figure 1. None of the foals developed hypoxemia, and the median SpO2 was 99.9 (97.1–100)% in the LIDO group and 98.6 (95–100)% in the HC group. The difference between the groups was statistically significant (*p* = 0.04). The mean F_E_’CO_2_ did not differ significantly between the two groups. The median F_E_’Iso was 1.1 (0.86–1.3)% in the LIDO group and 1.1 (0.9–1.3)% in the HC group without statistically significant differences between the two groups (*p* = 0.2). The cardiorespiratory parameters, the F_E_’CO_2_, and the F_E_’Iso of the foals of the two groups are shown in Table 3.

**Table 3:** Cardiorespiratory parameters and fraction of expired isoflurane (Fe’Iso) and of expired CO_2_ (F_E_’CO_2_) measured in foals undergoing general anesthesia and administered lidocaine intraoperatively (LIDO group) or not administered lidocaine intraoperatively (HC group).

Parameters	Groups	Baseline	Intraoperative	Recovery
HR (beats min^−1^)	LIDO	102.8 ± 22.8^a^	71.2 ± 13.4*^b^	84.6 ± 6.8^ab^
	HC	118.5 ± 24.6^a^	87.1 ± 17.7^b^	104.7 ± 27.9^ab^
		*p = 0.1*	*p = 0.02*	*p = 0.09*
MAP (mmHg)	LIDO	ND	69.8 ± 9.2	ND
	HC	ND	62.4 ± 9.7	ND
			*p = 0.08*	
SpO_2_ (%)	LIDO	ND	99.9 (97.1–100)*	ND
	HC	ND	98.6 (95–100)	ND
			*p = 0.04*	
F_E_’Iso (%)	LIDO	ND	1.1 (0.86–1.3)	ND
	HC	ND	1.1 (0.9–1.3)	ND
			*p = 0.2*	
F_E_’CO_2_ (mmHg)	LIDO	ND	48.5 ± 4.0	ND
	HC	ND	47.5 ± 7.3	ND
			*p = 0.73*	

Heart rate (HR) was measured at anesthetic evaluation (baseline) during general anesthesia (intraoperative) and at the recovery. The mean arterial pressure (MAP) the oxygen saturation (SpO_2_) and the fraction of expired isoflurane (F_E_’Iso) and of expired CO_2_ (F_E_’CO_2_) were measured intraoperatively. ND: not determined. Data are reported as mean ± standard deviation (SD). *Significantly (*p* < 0.05) different from the HC group. Data with different letters in a row (^a,b,c^) indicate a significant difference between each time point (*p* < 0.05).

Inotropic support with dobutamine was provided to 7 out of 12 foals in the LIDO group and 9 out of 11 foals in the CON group. The number of foals requiring inotropic support was not statistically associated with the treatment group (*p* = 0.371). In addition, the median dose of dobutamine did not differ significantly between the two groups (*p* = 0.6) and was 0.003 (0.0012–0.005) mg/kg/min in the LIDO group and 0.0025 (0.001–0.006.9) mg/kg/min in the HC group. In addition, two foals in the LIDO group and one foal in the HC group received additional norepinephrine intraoperatively (0.0005–0.001 mg/kg/min). Only one foal in the HC group received a ketamine bolus intraoperatively for inadequate intraoperative analgesia. The extubation time did not differ between the two groups, and it was 8.6 ± 7.1 min in the LIDO group and 7.7 ± 2.6 min in the HC group. The time to reach sternal recumbency was 20.6 ± 9.8 min in the LIDO group and 23.1 ± 11.3 min in the HC group. Both the extubation time and the time to reach sternal recumbency did not differ significantly between the two groups (*p* = 0.7 and *p* = 0.6, respectively).

The quality of recovery was recorded in five foals in the LIDO group and nine foals in the HC group. For all the foals in the HC group, the recovery was scored as 1, while in the LIDO group, one foal at the recovery was excited and was forcefully maintained into lateral recumbency, and therefore, the recovery was scored as 2. The median of the recovery score was 1 for both groups and did not differ significantly (*p* = 0.7).

One foal with intussusception in the LIDO group was euthanized for 15 h postoperatively for deterioration of the clinical condition. Another foal with intussusception of the same group died after 10 h postoperatively for development of pulmonary oedema. In the HC group, two foals were euthanized within a week postoperatively: one foal with uroperitoneum was euthanized 40 h after recovery for deterioration of the clinical condition and one foal with septic arthritis was euthanized after 5 days for worsening of the arthritis. All the other foals were discharged.

## 4 Discussion

In the present study, the anesthetic effects of lidocaine CRI in neonatal foals during general anesthesia were described for the first time. The results highlighted that lidocaine CRI did not provide a significant anesthetic sparing effect in isoflurane-anesthetized foals when administered at 0.05 mg/kg/min. The sparing effect of lidocaine on the requirement of inhalant anesthetic agents has been widely described in adult horses, providing a reduction of 25–26.7% of the inhalant anesthetic requirement (9, 10, 14). Sample size calculation of the present study was based on the assumption that a 20% reduction in the fraction of expired isoflurane would be significant. However, the assumption was not met by the actual data, and therefore, the study might be underpowered. In addition, previous studies have demonstrated that the sparing effect of lidocaine is dose dependent, and it is observed at serum concentrations obtained when a loading bolus of lidocaine is administered before commencing the CRI (14). In the present study, the lidocaine serum concentration was not evaluated and, since the pharmacokinetic parameters of drugs are affected by aging, especially the volume of distribution and clearance, the pharmacokinetic data obtained for lidocaine in adult horses cannot be applied to young foals (15). Furthermore, young foals are characterized by immaturity of the renal and hepatic systems (12), and by a lower plasmatic protein concentration; therefore, despite the increased volume of distribution, the rate of administration of drugs in these patients should be reduced to avoid reaching toxic plasmatic concentrations. Additional studies are warranted to describe the pharmacokinetics of lidocaine in anesthetized foals. Nannarone et al. (16) have previously observed that, in adult colic horses, the isoflurane sparing effect of lidocaine CRI does not differ based on whether a loading bolus is administered or not. Therefore, avoiding a loading dose of lidocaine in anesthetized horses might be safer, especially in unhealthy animals in which comorbidities might result in altering the disposition of the drugs when compared with healthy patients (16, 17). In experimental studies, 20–30 min of equilibrating period were observed before adjusting the isoflurane concentration (18), which was not the case of the present study and we cannot exclude that this decision had influenced the results. However, in foals, due to the high minute ventilation and CO, the uptake and the elimination of anesthetics via the lungs are more rapid if compared with adult animals (12). This same approach has already been described in adult horses (19), and it was taken into consideration to avoid prolonging the anesthesia time in neonatal patients at higher anesthetic risk.

In both groups, the HR decreased significantly intraoperatively, and it was still lower at recovery than that observed at baseline. Moreover, intraoperatively, the HR was significantly lower in the lidocaine-treated group. The effects of butorphanol on the HR in foals are negligible, but comparable HR values were reported in isoflurane anesthetized foals (13, 20), while studies regarding the cardiovascular effects of lidocaine in foals are lacking. In awake adult horses, lidocaine at high doses does not produce significant cardiovascular alterations (9, 16, 21). It can be also be hypothesized that lidocaine provided more effective analgesia when compared with butorphanol alone, accounting for the lower HR in the LIDO patients. The authors’ hypothesis is supported by previous studies investigating the analgesic effects of intravenous lidocaine (22–24). In fact, in ponies undergoing castration, electroencephalogram evaluation supported the anti-nociceptive effects of lidocaine given IV (19). The evaluation of intraoperative analgesia is challenging and is usually based on the evaluation of the alteration of the HR, *f*R, and MAP. However, since foals were mechanically ventilated, the evaluation of the *f*R was not taken into account for this purpose. In anesthetized foals, the maintenance of a physiologic HR is pivotal. In fact, the neonatal heart is characterized by few contractile elements and by prevalent connective tissues. Consequently, the foals’ heart is poorly compliant and characterized by a fixed stroke volume with a cardiac output (CO) depending essentially on the HR. This peculiarity associated with the immaturity of the sympathetic nervous system poses the foals at a higher risk of developing hypotension or hypoperfusion (1, 2, 12). In the absence of complete cardiovascular monitoring, the authors could not evaluate whether the decrease in the HR observed in the present cohort was associated with significant cardiovascular impairment; however, the addition of lidocaine infusion in anesthetized foals, despite the lower HR, was not associated with a higher incidence of hypotension. In fact, the mean arterial pressure did not differ between the two groups, and inotropic or vasoactive support was provided to some foals in both groups without significant differences. Episodes of hypotension are common in equine anesthesia, especially in ill neonatal patients (25), and therefore, continuous patient monitoring is advocated, with particular attention to the HR, blood pressure, and tissue perfusion.

There are controversies regarding the definition of hypotension in neonates. In our practice, as in the present study, it is common to support the patient by using inotropic drugs or vasopressors when the MAP is lower than 60 mmHg, since this has been advocated as a reference value to maintain an adequate tissue perfusion in neonates (2, 11).

Several authors have investigated the effects of lidocaine on the quality of recovery of adult equine patients (8–10, 16, 22). Even if lidocaine CRI has been associated with an uneventful and satisfactory recovery (9, 10, 22), some degrees of ataxia can be evident when lidocaine is administered intraoperatively (10, 26). In the present study, the overall quality of recovery was scored as smooth in both groups.

The inclusion of an historical control group was chosen with the aim of comparing the results of the treatment group not only to reduce the number of animals to be included but also to give to the patients prospectively included the treatment considered to be superior for anesthetic management (27). However, this choice might have underpowered the results obtained, and we cannot exclude different biases in the selection of animals. Among biases, we should also count that different anesthetists performed the anesthetic procedures, as required by the clinical nature of this study, and it is known that the anesthetic experience results in a more accurate evaluation of the anesthetic depth (28). Also, a foal in the HG group received ketamine intraoperatively to provide adequate analgesia, which may have further underpowered the results of the study but also highlight that, in the clinical setting, the continuous monitoring of the patient is essential for adjusting the analgesic protocol when needed. Further prospective and randomized studies are advocated for evaluating the effect of lidocaine in anesthetized foals with the support of a pharmacokinetic evaluation.

In conclusion, the results of the present study suggest that the addition of lidocaine CRI of 0.05 mg/kg/min in anesthetized foals does not provide a significant anesthetic sparing effect as well a significant effect on the cardiovascular parameters, and it does not interfere with the quality of recovery.

## Data availability statement

The raw data supporting the conclusions of this article will be made available by the authors without undue reservation.

## Ethics statement

The study has been assessed by the Animal Welfare Committee of the University of Bologna, which has provided a positive ethical-scientific evaluation and confirmed its compliance with national laws on the matter.

## Author contributions

LC: Data curation, Investigation, Formal analysis, Writing – original draft. SF: Data curation, Investigation, Writing – original draft. MA: Data curation, Investigation, Writing – original draft. SA: Data curation, Conceptualization, Writing – review & editing. LA: Data curation, Investigation, Writing – original draft. RN: Conceptualization, Data curation, Writing – review & editing, Investigation.
